# The time interval between laparoscopic tubal ligation and frozen-thawed embryo transfer does not affect the reproductive outcomes

**DOI:** 10.1186/s12884-023-05880-3

**Published:** 2023-08-03

**Authors:** Lijuan Fan, Xiaojuan Li, Juanzi Shi, Haixia Duan

**Affiliations:** 1 Assisted Reproduction Center, Northwest Women’s and Children’s Hospital, Xi’an, People’s Republic of China; 2 Department of Reproductive Gynecology, Northwest Women’s and Children’s Hospital, No. 73, Houzaimen Street, Xi’an, People’s Republic of China

**Keywords:** Hydrosalpinx, Laparoscopic tubal ligation, Frozen-thawed embryo transfer, Reproductive outcome, Neonate outcome

## Abstract

**Background:**

Hydrosalpinx may decrease implantation and pregnancy rates after embryo transfer. Laparoscopic tubal ligation after embryo freeze and before frozen-thawed embryo transfer (FET) is effective at improving reproductive outcomes for hydrosalpinx patients. This study is to find out the optimal interval between laparoscopic tubal ligation and FET.

**Methods:**

We retrospectively analyzed 259 infertile women who performed laparoscopic tubal ligation for embryo freeze and FET. Participants were divided into three groups, based on the interval between laparoscopic tubal ligation and FET. Group I: <30 days; Group II: 31– 60 days; Group III: >60 days. Outcomes of cleavage-stage and blastocyst-stage embryo FET were analyzed respectively.

**Results:**

There was no significant difference in clinical pregnancy rate, live birth rate, implantation rate, biochemical pregnancy rate, ectopic pregnancy rate, miscarriage rate and preterm birth rate among the three groups, in both cleavage-stage and blastocyst-stage embryo FET cycles. In cleavage-stage embryo FET cycle, singleton gestational age was significantly younger in group III (38.11 ± 2.28 weeks) compared with group I (39.29 ± 1.06 weeks, *P* = 0.001) and group II (38.96 ± 1.05, *P* = 0.026). Singleton birth weight was significantly heavier in group II (3.65 ± 0.32 Kg) compared with group I (3.38 ± 0.29 Kg, *P* = 0.001) and group III (3.35 ± 0.60 Kg, *P* = 0.004). Twin birth weight was significantly heavier in group III (2.72 ± 0.43 Kg) compared to group I (2.23 ± 0.67 Kg, *P* = 0.002). In blastocyst-stage embryo FET cycles, twin gestational age was significantly younger in group II (34.07 ± 3.18 weeks) compared with group I (35.56 ± 2.27 weeks, *P* = 0.049) and group III (36.50 ± 1.47 weeks, *P* = 0.005). Twin birth weight was significantly heavier in group III (2.71 ± 0.39 Kg) compared to group II (2.39 ± 0.67 Kg, *P* = 0.009).

**Conclusions:**

The duration of the interval between laparoscopic tubal ligation and FET does not affect the reproductive outcomes; however, it may affect the neonate outcomes to some extent.

## Introduction

A hydrosalpinx is a distally blocked, dilated and swelled fallopian tube that arises as a result of salpingitis, typically a residual effect of pelvic inflammatory disease. Hydrosalpinx is found in 10–30% of infertile couples and they are less likely to have successful in vitro fertilization (IVF). Hydrosalpinx is often associated with lower implantation and pregnancy rates, and an increased spontaneous miscarriage rate in IVF cycles [1]. Meta-analyses revealed that not only was there a 50% reduction in both implantation and pregnancy rates, the spontaneous miscarriage rate was doubled in patients with hydrosalpinx, compared with women with non-hydrosalpinx related infertility [2].

Improved pregnancy and delivery rates have been observed when hydrosalpinx patients undergo salpingectomy, salpingostomy, proximal tubal occlusion and tubal ligation before IVF [3]. Each treatment has its own merits and drawbacks. Current data strongly suggested that laparoscopic salpingectomy is effective at improving clinical and ongoing pregnancy rates for hydrosalpinx patients; and the UK National Institute for Health and Care Excellence (NICE) recommends performing salpingectomy, preferably by laparoscopy, before IVF treatment [4]. However, the ovarian function could be adversely impacted following salpingectomy due to interference with the ovarian blood flow. Laparoscopic proximal tubal ligation showed similar IVF outcomes when compared with laparoscopic salpingectomy [5, 6] and was widely recognized as an alternative treatment for hydrosalpinx before in vitro fertilization-embryo transfer (IVF-ET) [7].

Most studies compared tubal ligation with non-interventionist approach in order to demonstrate that the surgery could also improve IVF outcomes for patients with hydrosalpinx. However, there is insufficient data to determine the optimal period to perform embryo transfer after tubal ligation. While the reproductive outcomes may have shown to be better after laparoscopic tubal ligation, little is known of the role that time interval between laparoscopic tubal ligation and embryo transfer plays in affecting the outcome. This study aims to determine an ideal time interval between laparoscopic tubal ligation and embryo transfer that can lead to an improved outcome for hydrosalpinx patients.

## Materials and methods

### Study design and participants

A retrospective study on infertile women with hydrosalpinx who visited the Assisted Reproduction Center of Northwest Women’s and Children’s Hospital for IVF from January 2017 to December 2020 was undertaken. Women who had undergone IVF in the Center were included if they fulfilled the following inclusion criteria: (i) less than 40 years of age; (ii) BMI ≤ 33 Kg/m^2^; (iii) presence of unilateral or bilateral hydrosalpinx on pelvic scanning and confirmed by laparoscopy and (iv) conducted frozen-thawed embryo transfer (FET) after laparoscopic hydrosalpinx ligation surgery. Women were excluded if they had: (i) severe intrauterine adhesion; (ii) uterine morphological abnormities (including uterus duplex, unicornous uterus and uterine fibroids distorting the uterine cavity); (iii) endometriosis and (iv) decreased or diminished ovarian reserve (DOR).

### Controlled ovarian stimulation and vitrified cryopreservation

Stimulation protocols include GnRH agonist protocol, GnRH antagonist protocol and progestin-primed ovarian stimulation protocol (PPOS). Recombinant human chorionic gonadotropin (OVIDREL, Merck Serono) or GnRH-a (Decapeptyl, Ferring) were administered in patients if two leading follicle reached 18 mm. Oocyte retrieval was performed 36 h later after recombinant human chorionic gonadotropin or GnRH-a triggered by transvaginal ultrasonography guided aspiration. Insemination method was selected based on the sperm count of the prepared sperm. A morphologic score of cleavage-stage embryo was given based on the number of blastomeres, the homogeneous degree of blastomeres, and the degree of cytoplasmic fragmentation. The protocol was extensively described in our previous study [8]. If a couple has two or more good quality cleavage-stage embryos on day 3 of embryo culture, those embryos were selected for further blastocyst culture. Blastocyst evaluation was performed according to Gardner Grade Standard [9]. All the cleavage-stage embryos and blastocyst-stage embryos were cryopreserved by vitrification.

### Laparoscopic tubal ligation

After the embryos were cryopreserved, participants were offered laparoscopic tubal ligation before FET. After the surgeons had dilated the fallopian tube with methylene blue, unilateral or bilateral tubal ligation was performed for unilateral and bilateral hydrosalpinx patients respectively. During laparoscopy, tubal ligation was performed by bipolar diathermy applied at 1 to 1.5 cm away from the corneal section of the fallopian tube. Distal fenestration of the fallopian tube was carried out to release the fluid inside [10].

### Endometrial preparation and frozen-thawed embryo transfer

The interval between laparoscopic tubal ligation and FET depended on the patient’s personal decision. Patients generally underwent a natural or hormone replacement treatment (HRT) FET cycle. In natural FET cycles, cleavage embryo (or blastocyst) transfer was performed 4 (or 6) days after the LH surge. In HRT FET cycles, endometrial preparation was initiated with oral estradiol valerate (Progynova, Bayer) at a daily dose of 4 mg to 6 mg, starting from day 5 of the menstrual cycle. To reach an ideal endometrial thickness of ≥ 7 mm, some patients had to prolong their estrogen supplement duration to increase their endometrial thickness. The number of transferred embryos was determined by the conditions of all embryos and patient preferences. For all the participants, a maximum of two embryos were replaced.

Patients were divided into three groups according to the interval between laparoscopic tubal ligation and FET. Group I: the interval between laparoscopic tubal ligation to embryo transfer was less than 30 days; Group II: the interval was 31 to 60 days; Group III: the interval was more than 60 days.

### Luteal phase support and pregnancy confirmation

Luteal phase support was given using vaginal progesterone gel (90 mg q.d; Crinone, Serono, Hertfordshire, UK) and 20 mg dydrogesterone (Abbott, USA) on a daily basis. Biochemical pregnancies were diagnosed by screening for positive serum β-hCG 14 days after embryo transfer. Clinical pregnancy was confirmed by visualization of a gestational sac via ultrasonographic examination 5 weeks after the embryo transfer [11]. Luteal support treatment was provided up to 10 weeks for confirmed pregnancies. All pregnant women were contacted or traced for the pregnancy outcomes after delivery or miscarriage.

### Outcomes

We used the American Society for Reproductive Medicine (ASRM) 2017 consensus definitions [12] for defining the clinical outcomes. The primary outcomes were the clinical pregnancy rate and live birth rate. The secondary outcomes included implantation rates, biochemical pregnancy rate, ectopic pregnancy rate, miscarriage rate, preterm birth rate and neonate outcomes. The implantation rate was calculated as the number of gestational sacs seen on scanning divided by the number of embryos replaced. The biochemical pregnancy rate was calculated as the number of pregnancies diagnosed by the detection of β-hCG in serum per woman after embryo transfer. Ectopic pregnancy described a pregnancy outside the uterine cavity, diagnosed by ultrasound, surgical visualization or histopathology. The miscarriage rate was defined as the number of intra-uterine pregnant loss before 22 weeks divided by the number of women who performed FET. Preterm delivery was defined as delivery that occurred between 22 weeks and 37 weeks of gestational age. The follow-up contents of neonate outcomes included birth weight, gestation age and birth defects.

### Statistical analysis

The descriptive data on participant characteristics were summarized using the mean and standard deviation for continuous variables; counts and proportions were used for the categorical variables. Chi-squared tests or Fisher exact tests were performed to compare the categorical variables. To compare continuous variables among the groups, an analysis of covariance was used. Logistic regression was performed to reduce potential bias caused by the number of embryo transfer on FET outcome, presenting the odds ratio (OR) and corresponding 95% confidence interval (CI) to compare FET outcome. All statistical analyses were executed with SPSS 23.0 software package. *P* values were two-sided with a significance level of < 0.05.

## Results

### Baseline characteristics of the three groups

During the study period, 259 patients fulfilled the inclusion criteria and were analyzed. The patients’ distribution (based on the interval between laparoscopic tubal ligation and FET) was as follows: group I (115 patients, 44.79%), group II (82 patients, 31.66%), and group III (62 patients, 23.94%). Patient baseline characteristics were summarized in Table 1. The three groups were similar in age at oocyte pick up (OPU), infertility duration, BMI, basal FSH, basal LH, hydrosalpinx laterality, duration of ovarian stimulation, total units of gonadotropin usage, number of retrieved oocytes, HRT/natural transfer cycle ratio, endometrial preparation duration and endometrial thickness (*P* > 0.05).

**Table 1:** Baseline characteristics, controlled ovarian stimulation parameters and FET parameters of the three group

Variables	Group I (n = 115)	Group II (n = 82)	Group III (n = 62)	*P* value
Patients age at OPU (y)	30.19 ± 4.00	31.4 ± 3.55	30.94 ± 3.45	0.075
Infertility duration (y)	3.78 ± 2.87	4.05 ± 3.21	4.15 ± 2.90	0.700
BMI (Kg/m^2^)	22.02 ± 2.87	22.11 ± 3.25	22.90 ± 3.59	0.184
Basal FSH (IU/L)	7.68 ± 2.49	7.91 ± 2.21	7.73 ± 2.88	0.814
Basal LH (IU/L)	4.34 ± 2.08	4.37 ± 1.99	4.41 ± 2.05	0.977
Hydrosalpinx				
Bilateral hydrosalpinx (%)	70.43 (81/115)	57.50 (46/80)	57.89 (33/57)	0.111
Unilateral hydrosalpinx (%)	29.57 (34/115)	42.50 (34/80)	42.11(24/57)	
Duration of ovarian stimulation (d)	10.33 ± 2.37	10.49 ± 2.26	10.02 ± 2.43	0.485
Units of Gn (IU)	2390.33 ± 848.90	2396.05 ± 841.96	2172.98 ± 793.15	0.196
NO. of retrieved oocytes	11.21 ± 5.78	11.21 ± 5.40	11.15 ± 6.57	0.997
FET cycle				
HRT transfer cycle (%)	85.22 (98/115)	90.00 (72/80)	94.74 (54/57)	0.162
Natural transfer cycle (%)	14.78 (17/115)	10.00(8/80)	5.26 (3/57)	
Endometrial preparation duration (d)	11.03 ± 3.50	11.77 ± 3.73	11.32 ± 3.12	0.340
Endometrial thickness (mm)	10.23 ± 1.49	10.05 ± 1.39	10.12 ± 1.10	0.649

Continuous variables are presented as mean ± standard deviation, categorical variables are presented as number (percentage). *OPU* oocyte pick up; *BMI* body mass index; *FSH* follicle stimulating hormone; *LH* luteinizing hormone; *Gn* gonadotropin; *FET* frozen-thawed embryo transfer

### Reproductive and perinatal outcomes after FET

Considering that there are differences in obstetrical and perinatal outcomes observed between blastocyst-stage embryo FET and cleavage-stage embryo FET [13], we compared cleavage-stage embryo FET outcomes and blastocyst-stage FET outcomes individually. Logistic regression was performed to adjust for potential confounder.

The reproductive and neonate outcomes from cleavage-stage embryo FET were presented in Table 2. No significant differences were found among the three groups in reproductive outcomes, including implantation rate, biochemical pregnancy rate, clinical pregnancy rate, ectopic pregnancy rate, miscarriage rate, live birth rate, preterm birth rate and twin birth rate. In singleton birth cleavage-stage embryo FET, gestational age at birth of group III (38.11 ± 2.28 weeks) was significantly younger when compared with group I (39.29 ± 1.06 weeks, *P* = 0.001) and group II (38.96 ± 1.05 weeks, *P* = 0.026). Singleton birth weight was significantly heavier in group II (3.65 ± 0.32 Kg) compared with group I (3.38 ± 0.29 Kg, *P* = 0.001) and group III (3.35 ± 0.60 Kg, *P* = 0.004). No birth defects were found in singletons for all three groups. Twin birth weight was significantly heavier in group III (2.72 ± 0.43 Kg) compared to group I (2.23 ± 0.67 Kg, *P* = 0.002). One of the twins in group I was diagnosed with duodenal atresia.

**Table 2:** Reproductive and neonate outcomes of cleavage-stage embryo FET

Variables	Group I (n = 68)	Group II (n = 49)	Group III (n = 34)	OR group II VS group I group III VS group I	95%CI group II VS group I group III VS group I	*P* value
NO. of cleavage-stage embryo transfers	1.94 ± 0.24	1.89 ± 0.31	1.98 ± 0.13	-	-	0.098
Double cleavage-stage embryo transfer ratio (%)	94.12 (64/68)	87.76 (43/49)	97.06 (33/34)	-	-	0.231
Total embryo transfer	132	92	67	-	-	-
High quality cleavage-stage embryo ratio (%)	61.36 (81/132)	54.35 (50/92)	50.75 (34/67)	-	-	0.310
Implantation rate (%)	44.70 (59/132)	38.04 (35/92)	41.79 (28/67)	-	-	0.611
Biochemical pregnancy rate (%)	69.12 (47/68)	63.27 (31/49)	61.76 (21/34)	1.408 1.119	0.593, 3.342 0.449, 2.791	0.708
Clinical pregnancy rate (%)	67.65 (46/68)	59.18 (29/49)	61.76 (21/34)	1.309 0.929	0.554, 3.094 0.376, 2.297	0.652
Ectopic pregnancy rate (%)	4.41 (3/68)	2.04 (1/49)	0.00 (0/34)	0.998 0.998	0.000 0.000	0.970
Miscarriage rate (%)	8.82(6/68)	10.20(5/49)	5.88 (2/34)	1.841 1.834	0.361, 9.395 0.331, 10.164	0.744
Live birth rate (%)	54.41 (37/68)	46.94 (23/49)	55.88 (19/34)	0.948 0.713	0.414, 2.174 0.294, 1.730	0.685
Preterm birth rate (%)	7.35 (5/68)	8.16 (4/49)	8.82 (3/34)	0.667 1.026	0.140, 3.172 0.312, 4.934	0.812
Twin birth rate (%)	16.18 (11/68)	16.33 (8/49)	11.76 (4/34)	0.515 0.445	0.440, 5.151 0.453, 6.064	0.736
Singleton neonates	n = 26	n = 15	n = 15			
Gestational age at birth (weeks)	39.29 ± 1.06^a^	38.96 ± 1.05^b^	38.11 ± 2.28^ab^			0.003
Birth weight (Kg)	3.38 ± 0.29^c^	3.65 ± 0.32^bc^	3.35 ± 0.60^b^			0.001
Birth defect ratio (%)	0.00 (0/26)	0.00 (0/15)	0.00 (0/15)			
Twin neonates	n = 22	n = 16	n = 8			
Gestational age at birth (weeks)	34.84 ± 2.87	35.05 ± 2.50	36.32 ± 1.89			0.396
Birth weight (Kg)	2.23 ± 0.67^a^	2.43 ± 0.59	2.72 ± 0.43^a^			0.007
Birth defect ratio (%)	4.55 (1/22)	0.00 (0/16)	0.00 (0/8)			

*CI* confidential interval; *OR* odds ratio; *Exact fisher; ^a^*P*<0.05 (group I vs. group III); ^b^*P*<0.05 (group II vs. group III); ^c^*P*<0.05 (group I vs. group II)

The reproductive and perinatal outcomes after blastocyst-stage embryo FET were presented in Table 3. No significant differences in reproductive outcomes were found within the three groups. No ectopic pregnancy occurred in the three groups. In singleton birth after blastocyst-stage embryo FET, there were no significant differences in gestational age at birth or birth weight. In twin birth, gestation age was significantly younger in group II (34.07 ± 3.18 weeks) compared with group I (35.56 ± 2.27 weeks, *P* = 0.049) and group III (36.50 ± 1.47 weeks, *P* = 0.005). Twin birth weight was significantly heavier in group III (2.71 ± 0.39 Kg) compared to group II (2.39 ± 0.67 Kg, *P* = 0.009).

**Table 3:** Reproductive and neonate outcomes of blastocyst-stage FET

Variables	Group I (n = 42)	Group II (n = 33)	Group III (n = 28)	OR group II VS group I group III VS group I	95%CI group II VS group I group III VS group I	*P* value
NO. of blastocyst-stage transfers	1.71 ± 0.46	1.53 ± 0.50	1.78 ± 0.42	-	-	0.021
Double blastocyst-stage embryo transfer ratio (%)	71.43 (30/42)	51.52 (17/33)	75.00 (21/28)	-	-	0.098
Total blastocyst- stage transfer	72	50	49	-	-	-
High quality blastocyst ratio (%)	91.67 (66/72)	80.00 (40/50)	77.55 (38/49)	-	-	0.070
Implantation rate (%)	61.11 (44/72)	74.00 (37/50)	61.22 (30/49)	-	-	0.278
Biochemical pregnancy rate (%)	78.57 (33/42)	78.79 (26/33)	75.00 (21/28)	1.507 2.319	0.428, 5.308 0.591, 9.103	0.483
Clinical pregnancy rate (%)	78.57 (33/42)	78.79 (26/33)	71.43 (20/28)	1.965 3.158	0.553, 6.989 0.787, 12.669	0.265
Ectopic pregnancy rate (%)	0.00 (0/42)	0.00 (0/33)	0.00 (0/28)	-	-	-
Miscarriage rate (%)	9.52 (4/42)	3.03 (1/33)	7.14 (2/28)	1.447 0.501	0.244, 8.585 0.042, 5.999	0.646
Live birth rate (%)	69.05 (29/42)	72.73 (24/33)	64.29 (18/28)	1.451 2.519	0.484, 4.348 0.738, 8.598	0.332
Preterm birth rate (%)	13.79 (4/29)	16.67 (4/24)	7.14 (2/28)	1.480 2.522	0.248, 8.843 0.407, 15.640	0.587
Twin rate (%)	34.48 (10/29)	29.17 (7/24)	28.57 (8/28)	0.841 0.982	0.271, 2.615 0.281, 3.430	0.946
Singleton neonates	n = 19	n = 17	n = 10			
Gestation age (weeks)	38.47 ± 3.08	39.26 ± 0.87	38.11 ± 0.96			0.075
Birth weight (Kg)	3.50 ± 0.79	3.55 ± 0.41	3.27 ± 0.45			0.176
Birth defect ratio (%)	0.00 (0/19)	0.00 (0/17)	0.00 (0/10)			
Twin neonates	n = 20	n = 14	n = 16			
Gestation age (weeks), mean + SD	35.56 ± 2.27^c^	34.07 ± 3.18^bc^	36.50 ± 1.47^b^			0.016
Birth weight	2.51 ± 0.46	2.39 ± 0.67^b^	2.71 ± 0.39^b^			0.029
Birth defect ratio (%)	0.00 (0/20)	7.14 (1/14)	0.00 (0/16)			

Continuous variables are presented as mean ± standard deviation, categorical variables are presented as number (percentage). *CI* confidential interval; *OR* odds ratio; ^b^*P*<0.05 (group II vs. group III); ^c^*P*<0.05 (group I vs. group II)

## Discussion

The present study shows that there is no significant difference in clinical pregnancy rate or live birth rate among the different intervals between laparoscopic tubal ligation and FET. Implantation rates, biochemical pregnancy rates, ectopic pregnancy rates, miscarriage rates, preterm birth rates and twin birth rates were similar among the three groups as well. This indicates that the prolonged interval between hydrosalpinx tubal ligation and FET did not improve reproductive outcomes.

There are some significant differences in neonate outcomes among the groups. In the cleavage-stage embryo FET cycles, singleton gestation age was significantly younger in group III as compared to the other groups, and singleton birth weight was significantly heavier in group II as compared to group I and group III. In the blastocyst-stage embryo FET cycles, twin gestation age was significantly younger in group II as compared to the other two groups. However, the mechanisms causing the difference in neonate outcomes are unclear. There is currently no report on the effects of hydrosalpinx on neonate outcomes.

There is still a lack of understanding on how hydrosalpinx exerts its negative effects. The main theories proposed are focused on the impairment of endometrial receptivity. The crosstalk between the embryo and the endometrium may be disrupted due to leakage of hydrosalpinx fluid in the uterine cavity. Microorganisms, debris, leukocytes, cytokines, prostaglandins, and leukotrienes in the hydrosalpinx fluid could have affected the implantation process. Cytokines including HOXA10, leukemia inhibitory factor and integrin α_ν_β_3_ are all factors that have been shown to be of importance to implantation. The presence of hydrosalpinx may reduce endometrial receptivity by diminishing the expression of HOXA10, leukemia inhibitory factor and integrin α_ν_β_3_ [14]. Other theories have included simultaneous damage to the embryo. Bedaiwy et al. [15] showed the presence of reactive oxygen species in hydrosalpinx fluid and a possible role of oxidative stress leading to embryotoxicity was suggested. By using an in vitro embryo culture model, Spandorfer et al. [16] found that hydrosalpinx fluid could impede embryogenesis. Also, hydrosalpinx fluid may have adverse effects on germ cells. In a study on gametes and fertilization, one out of four hydrosalpinx fluids was found to be cytotoxic to murine spermatozoa when incubated in 50% hydrosalpinx during capacitation [17].

Surgical treatment of tubal disease prior to IVF is beneficial for increasing the pregnancy and live birth rate. The following surgical treatments for tubal disease were considered: salpingectomy, tubal occlusion, salpingostomy and hydrosalpinx ligation. Each treatment has its own merits and drawbacks. Salpingectomy comes with the advantage of having the chronically infected tissue removed totally, thus eliminating the risk of abscess formation or torsion and increasing the accessibility of the ovary during oocyte retrieval in IVF. Drawbacks, however, related to the invasiveness of the procedure itself and the difficult procedure required when there are dense adhesions. Furthermore, it has been suggested that salpingectomy may affect ovarian function by interfering with ovarian blood flow [18]. Proximal tubal occlusion with Essure® devices placed hysteroscopically can be considered for specific cases of distorted pelvic anatomy or pelvic adhesions. However, low clinical pregnancy and live birth rates have been reported from the use of tubal occlusion devices [10]. Salpingostomy was thought to have the advantage of being less invasive and safer than salpingectomy while allowing the woman to attempt natural conception. The disadvantages mentioned include a possibility of hydrosalpinx recurrence and a high risk of ectopic pregnancy rate (10%) [19].

Chu et al. [19] reported a high risk of ectopic pregnancy at 10% after hydrosalpinx salpingostomy. Salpingectomy also carries an increased risk of interstitial pregnancy [3]. Wang et al. identified 43 cases of interstitial pregnancy, of which 71% had undergone bilateral salpingectomy before IVF [20]. In our present study, the ectopic pregnancy rate after hydrosalpinx ligation was 0–4.1% in cleavage-stage embryo FET cycles, with a decrease in ectopic pregnancy incidence over time. In our blastocyst-stage embryo FET cycles, none of the patients suffered ectopic pregnancy. This suggests that ectopic pregnancy rate may be decreased by delaying cleavage-stage embryo FET or by transferring blastocyst-stage embryos.

The advantage of proximal tubal ligation over salpingectomy is that this surgery can easily be performed and it does not damage ovarian reserve as compared to salpingectomy. In our center, we combined laparoscopic proximal tubal ligation with salpingostomy to reduce the damage of hydrosalpinx on adjacent organs and tissues.

We like to highlight the strong points in our present study: First, we followed up with the participants after delivery or miscarriage. The data of neonates was well recorded and analyzed in our present study. Generally, in earlier publications of this topic, neonate outcomes after hydrosalpinx tubal ligation were not analyzed. Second, our sample size was large; a total of 259 infertile women who performed laparoscopic tubal ligation between embryo freeze and FET were included. Besides, the clinical pregnancy rates in this study were also higher than that of the previous studies [6]. Finally, this study is a single-center cohort study, in which the practice can be assured to be consistent.

We also acknowledge several limitations of this study. We only compared female age at OPU among the three groups, without comparing their age at FET. For 76.06% of the participants who underwent FET in two months after laparoscopic tubal ligation, the time interval between OPU and FET was short.

## Conclusions

In conclusion, the interval between laparoscopic tubal ligation and FET does not affect reproductive outcomes of hydrosalpinx patients, but it may affect neonate outcomes to some extent. Furthermore, multi-center prospective randomized clinical trials will be needed to confirm the effects of the different intervals between laparoscopic tubal ligation and FET on reproductive outcomes and neonate outcomes. This study will aid in patients who are seeking hydrosalpinx counseling for FET after laparoscopic tubal ligation.

## Data Availability

The datasets used and analysed during the current study are available from the corresponding author on reasonable request.

## Abbreviations

FET: Frozen-thawed embryo transfer
IVF: In vitro fertilization
IVF-ET: In vitro fertilization-embryo transfer
NICE: National Institute for Health and Care Excellence
DOR: Diminished ovarian reserve
PPOS: Progestin-primed ovarian stimulation protocol
HRT: Hormone replacement treatment
ASRM: American Society for Reproductive Medicine
